# Decreased interleukin 27 expression is associated with active uveitis in Behçet’s disease

**DOI:** 10.1186/ar4570

**Published:** 2014-05-28

**Authors:** Chaokui Wang, Yuan Tian, Zi Ye, Aize Kijlstra, Yan Zhou, Peizeng Yang

**Affiliations:** 1 First Affiliated Hospital of Chongqing Medical University, Chongqing Key Lab of Ophthalmology, Chongqing Eye Institute, Youyi Road 1, Chongqing 400016, People’s Republic of China; 2 University Eye Clinic Maastricht, P. Debyelaan 25, 6229 HX Maastricht, The Netherlands

## Abstract

**Instruction:**

Interleukin 27 (IL-27) is an important regulator of the proinflammatory T-cell response. In this study, we investigated its role in the pathogenesis of Behçet’s disease (BD).

**Methods:**

IL-27 mRNA in peripheral blood mononuclear cells (PBMCs) was examined by performing RT-PCRs. Cytokine levels in sera or supernatants of PBMCs, naïve CD4^+^ T cells, dendritic cells (DCs) and DC/T cells were determined by enzyme-linked immunosorbent assay. We used RNA interference in naïve CD4^+^ T cells to study the role of interferon regulatory factor 8 (IRF8) in the inhibitory effect of IL-27 on Th17 cell differentiation. Flow cytometry was used to evaluate the frequency of IL-17- and interferon γ–producing T cells.

**Results:**

The expression of IL-27p28 mRNA by PBMCs and IL-27 in the sera and supernatants of cultured PBMCs were markedly decreased in patients with active BD. A higher frequency of IL-17-producing CD4^+^ T (Th17) cells and increased IL-17 production under Th17 polarizing conditions were observed in patients with active BD. IL-27 significantly inhibited Th17 cell differentiation. Downregulation of IRF8 by RNA interference abrogated the suppressive effect of IL-27 on Th17 differentiation. IL-27 inhibited the production of IL-1β, IL-6 and IL-23, but promoted IL-10 production, by DCs. IL-27-treated DCs inhibited both the Th1 and Th17 cell responses.

**Conclusions:**

The results of the present study suggest that a decreased IL-27 expression is associated with disease activity in BD patients. Low IL-27 expression may result in a higher Th1 and Th17 cell response and thereby promote the autoinflammatory reaction observed in BD. Manipulation of IL-27 may offer a new treatment modality for this disease.

## Introduction

Behçet’s disease (BD) is an autoinflammatory disease characterized by ocular, mucosal, skin and neurological lesions. It is one of the most common uveitis entities seen in the countries along the old Silk Road, such as Japan, Turkey, Israel and China [1,2]. The pathogenesis of BD is unclear, and it is currently recognized as an autoinflammatory disease in which environmental triggers initiate an aberrant inflammatory response in genetically susceptible hosts [3,4]. Researchers in previous studies have shown that a type 1 T helper (Th1) cell response may play an important role in the pathogenesis of this disease. Increased expression of Th1-associated cytokines such as interferon γ (IFN-γ) and interleukin 12 (IL-12) has been documented in BD patients [5,6]. However, in recent studies, we and others have provided convincing evidence that the IL-23/IL-17 pathway is also involved [7,8]. Researchers who have conducted studies in mice have reported that strategies aimed at suppressing the Th17 cell response were an effective therapeutic approach in experimental autoimmune uveitis (EAU), a mouse model of uveitis [9].

IL-27, a heterodimeric cytokine composed of two subunits: p28 (IL-27p28) and the Epstein-Barr virus–induced gene 3 (*EBI3*), is produced mainly by activated antigen-presenting cells (APCs) [10]. The IL-27 receptor is widely expressed on naïve T cells, natural killer cells, mast cells, monocytes, keratinocytes, vascular endothelium, activated B cells, DCs and Langerhans cells [11]. Researchers in previous studies have shown that IL-27 is a proinflammatory cytokine which can induce Th1 differentiation [12]. Recently, however, researchers have shown that IL-27 has immunosuppressive properties that can suppress experimental autoimmune encephalomyelitis (EAE) and EAU by inhibiting the development or expansion of Th17 cells and inducing type 1 regulatory T (Tr1) cells [7,13,14].

Intraocular inflammation in BD often leads to blindness in spite of treatment with immunosuppressive drugs [15]. A search has therefore started to develop alternative treatment options. Biologic therapy with IFN-α has recently been introduced, with promising results [16]. The mode of action of IFN-α has not yet been elucidated, but one of its potential mechanisms lies in its ability to augment immunoregulatory IL-27 production [17]. In view of these findings, we decided to investigate whether IL-27 production might be altered in patients with BD. The results of preliminary studies conducted at the National Eye Institute (National Institutes of Health, Bethesda, MD, USA) [7,18] have suggested that IL-27 might play a role in controlling the aberrant Th17 response in uveitis, which provided us with a further rationale for undertaking the study described here.

## Methods

### Patients

We included 19 patients with active BD (10 men and 9 women, with an average age of 34.5 years) and 20 patients with inactive BD (11 men and 9 women, with an average age of 35.8 years) in this study. Twenty-eight healthy individuals (15 men and 13 women, with an average age of 36 years) acted as controls. The detailed demographics of the patients are provided in Additional file 1. We used the diagnostic criteria developed by the International Study Group for Behçet’s Disease [19,20]. We defined active intraocular inflammation on the basis of the presence of decreased vision (100%), keratic precipitates (100%), anterior chamber flare and cells (100%), cells in the vitreous (81%) and retinal vasculitis (100%). We assessed retinal vasculitis by fundus fluorescein angiography. The 19 BD patients with active uveitis were enrolled into this study on their first visit to our hospital. Fourteen of these patients had not used any immunosuppressive agents before visiting us, due either to not being referred to a hospital or to their worry about the side effects of systemic corticosteroids. The other five patients had previously been given low-dose systemic corticosteroids (<20 mg/day), but they had stopped using the drugs for about half a month at the time they visited our institution and were sampled of peripheral blood. We normally treat patients with BD by prescribing systemic corticosteroids in combination with cyclosporine, cyclophosphamide or chlorambucil for more than 1.5 years. The drug dose is gradually tapered after intraocular inflammation is under control, and the treatment is usually stopped 6 months after complete control of the intraocular inflammation. After all medications had been stopped for at least 2 months, we collected blood samples from the patients with inactive BD.

Written informed consent to participate in the study was received from all patients and healthy controls. Our study was designed to adhere to the tenets of the Declaration of Helsinki, and the study protocol was approved by the Clinical Ethical Research Committee of our medical faculty.

### Cell cultures and human Th17 cell polarization

We collected heparinized blood from our patients, and peripheral blood mononuclear cells (PBMCs) were subsequently isolated by Ficoll-Hypaque density gradient centrifugation. The production of IL-27 by cultured PBMCs was investigated with or without *Staphylococcus aureus* Cowan Strain I (SAC) (0.02% concentration; Sigma-Aldrich, St Louis, MO, USA) at a density of 2 × 10^6^ cells/ml for 72 hours. Naïve CD4^+^ T cells were purified from PBMCs by negative selection using human naïve CD4 microbeads according to the manufacturer’s instructions (Miltenyi Biotec, Palo Alto, CA, USA, the purity of isolated naïve CD4+T cells in the study was >90%). Naïve CD4^+^ T cells (1 × 10^6^/ml) were cultured in 96-well plates in the presence or absence of recombinant human IL-27 (rIL-27) (100 ng/ml; R&D Systems, Minneapolis, MN, USA). For Th17 polarization, the following antibodies and cytokines were added at the time of plating: anti-CD3 and anti-CD28 (2 μg/ml) (eBioscience, San Diego, CA, USA), anti-IL-1β (10 ng/ml), anti-IL-23 (10 ng/ml), anti-IL-6 (50 ng/ml), anti-IFN-γ (10 μg/ml) and anti-IL-4 (10 μg/ml). IL-2 (100 ng/ml), which plays a critical role in the survival and physiology of the Th17 subset [7,21], was added on day 3. The antibodies and recombinant cytokines used in our Th17 polarization experiments were obtained from R&D Systems. After being cultured for 7 days, the supernatants were collected, followed by IL-17 protein quantification by enzyme-linked immunosorbent assay (ELISA) (R&D Systems). The cells were obtained for intracellular cytokine detection by flow cytometry. Experiments with patient cells were performed on different occasions, and often one or two patients and corresponding controls were included in a given experiment.

### Knockdown with small interfering RNA

To knock down the expression of interferon regulatory factor 8 (IRF8), naïve CD4^+^ T cells were purified and nucleoporated with a negative control (QIAGEN, Valencia, CA, USA) and IRF8 small interfering RNA (siRNA) using an Amaxa nucleoporator system (Lonza, Basel, Switzerland) according to procedures described previously [22]. Next, the cells were incubated at 37°C for 4 hours and then stimulated with or without human rIL-27 (100 ng/ml) under Th17 polarizing conditions for another 48 hours. The production of IL-17 in the collected culture supernatants was detected by ELISA, and the intracellular expression of IL-17 was determined by flow cytometry.

### Human dendritic cell culture

In order to study human dendritic cells (DCs), we cultured CD14^+^ monocytes (purity >90%; Miltenyi Biotec) in medium supplemented with human rIL-4 (50 ng/ml; R&D Systems) and recombinant human granulocyte-macrophage colony-stimulating factor (GM-CSF) (100 ng/ml; R&D Systems) at a density of 1 × 10^6^ as described previously [23]. On the sixth day of culture, lipopolysaccharide (LPS) (100 ng/ml) was added as a stimulus in combination with, or without, rIL-27 (100 ng/ml). After 48 hours, supernatants were collected for the measurement of IL-1β, IL-6, IL-23 and IL-10 by ELISA.

### Dendritic cell/T-cell coculture assay

CD4^+^ T cells were enriched by immunomagnetic selection (purity >93%). They were then cocultured with DCs that had been activated with LPS (100 ng/ml) with or without IL-27 (100 ng/ml) for 24 hours. DCs were washed three times and then cocultured with T cells at a 1:5 ratio of DCs to T cells in 96-well plates for a period of 4 days. The IL-17 level in culture supernatants was then measured by ELISA. The cells were harvested and tested for the presence of intracellular IL-17 and IFN-γ using flow cytometry.

### Flow cytometric analysis

For intracellular detection of cytokines, the cells were stimulated by adding 50 ng/ml phorbol 12-myristate 13-acetate (Sigma-Aldrich) and 1 μg/ml ionomycin (Sigma-Aldrich) for 1 hour at 37°C. Next, 10 μg/ml brefeldin A (Sigma-Aldrich) was added, and the cells were incubated for another 4 hours. The cells were subsequently fixed and permeabilized using a BD Cytofix/Cytoperm Kit according to the protocols supplied by the manufacturer (BD Biosciences, San Jose, CA, USA). The cells were then stained with fluorescent antibodies (anti-human CD8-APC, anti-human CD3-PerCP-Cy5.5 (CD3–peridinin chlorophyll–cyanine 5.5), anti-human IFN-γ-fluorescein isothiocyanate and anti-human IL-17A-phycoerythrin; all from BD Biosciences, Sunnyvale, CA, USA). Samples were analyzed on a FACScan flow cytometer and analyzed using CellQuest software (BD Biosciences).

### Real-time quantitative RT-PCR

Human naïve CD4^+^ T cells were polarized to Th17 cells as described above. RNA was isolated from PBMCs or polarized Th17 cells using an RNeasy Mini Kit (QIAGEN) according to the manufacturer’s protocols. cDNA was then synthesized using Superscript III Reverse Transcriptase (Invitrogen, Carlsbad, CA, USA). Real-time quantitative PCRs was were carried out on an Applied Biosystems 7500 Fast Real-Time PCR System (Applied Biosystems, Foster City, CA, USA) with the QuantiTect SYBR Green PCR Kit from the same manufacturer. The following primer sequences were used: *β-actin*: forward, 5′-GGATGCAGAAGGAGATCAC TG-3′, reverse, 5′-CGATCCACACGGAGTACTTG-3′; and *IRF8*: forward, 5′-GAAGACGA GGGTTACGCTGTG-3′, reverse, 5′-TCCTCAGGAACAATTCGGTAA-3′. To investigate the mRNA expression of the two subunits of IL-27, EBI3 and p28 were purchased from QIAGEN. Gene expression was normalized relative to the expression of β-actin using the comparative threshold cycle method [24].

### Statistical analysis

One-way analysis of variance, paired-samples *t-*test, Kruskal–Wallis test and Mann–Whitney *U* test were applied using SPSS version 12.0 statistical software (SPSS, Chicago, IL, USA). Data are expressed as mean ± standard deviation. Any difference with *P* < 0.05 was considered statistically significant. A Bonferroni correction was performed for multiple comparisons, and *P*_c_ < 0.05 was considered statistically significant.

## Results

### Decreased expression of interleukin 27p28 mRNA in PBMCs from patients with active BD

**Figure 1:**
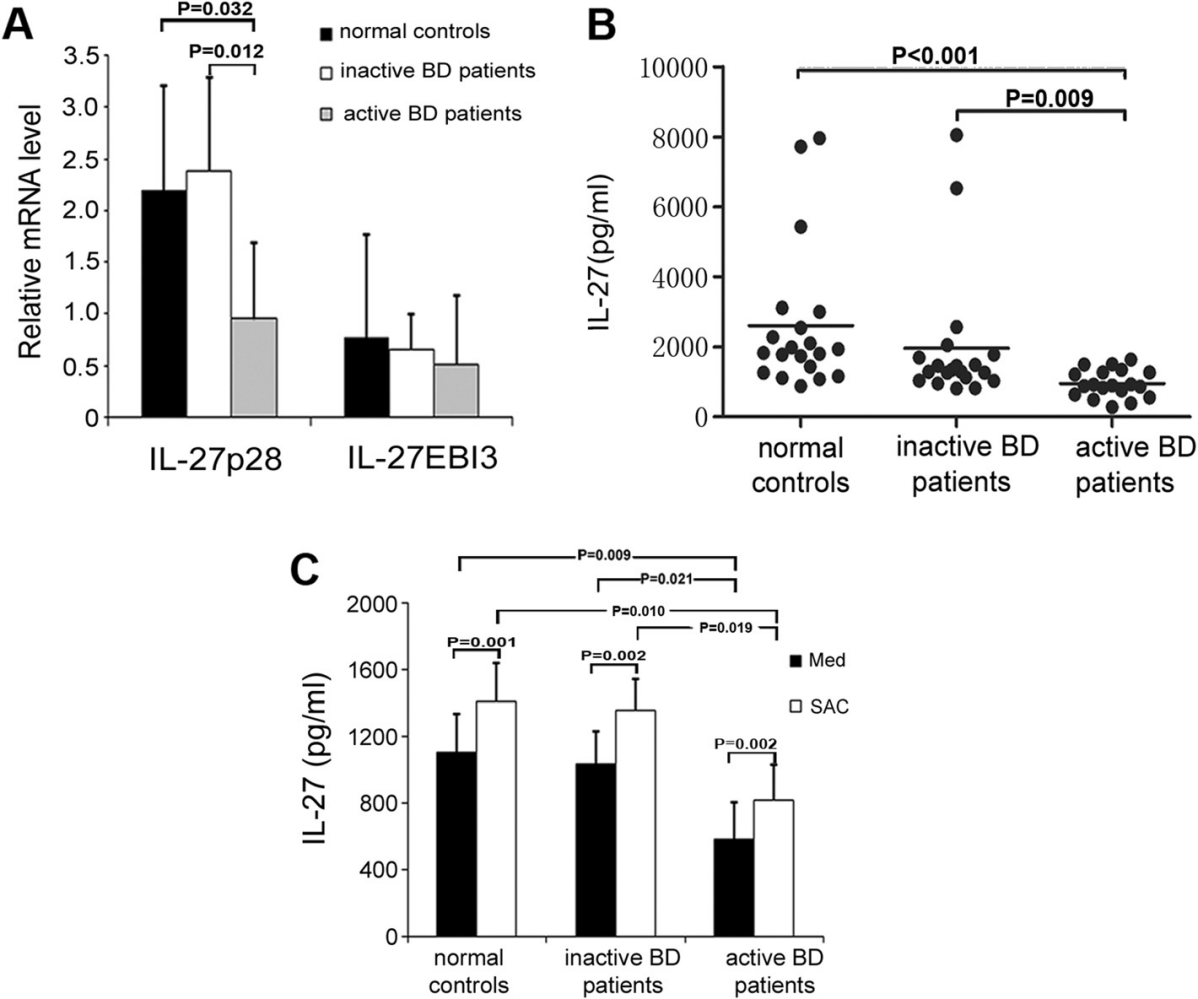
**Decreased level of interleukin 27 in patients with active Behçet’s disease. (A)** Relative gene expression of the interleukin (IL) subunits p28 and EBI3 in peripheral blood mononuclear cells (PBMCs) from patients with active Behçet’s disease (BD) (*n* = 8), patients with inactive BD (*n* = 8) and healthy controls (*n* = 8) was measured by real time PCR. The results of these experiments are presented as expression relative to β-actin. Analysis of variance (ANOVA) was used for statistical analysis. **(B)** IL-27 levels in sera from patients with active BD (*n* = 19), patients with inactive BD (*n* = 20) and healthy controls (*n* = 20) were assessed by enzyme-linked immunosorbent assay. Kruskal–Wallis and Mann–Whitney *U* tests were carried out for statistical analyses, followed by the Bonferroni correction (*P*_c_) for comparison of the three groups. *P*_c_ *<* 0.05 was considered significant. **(C)** IL-27 production by PBMCs from patients with active BD (*n* = 6), patients with inactive BD (*n* = 6) and healthy controls (*n* = 6) cultured with or without *Staphylococcus aureus* Cowan Strain I (SAC) was examined. A paired-samples *t-*test for related samples and one-way ANOVA for independent samples were performed for statistical analysis. The Bonferroni correction was used to correct for multiple comparisons. The statistical significance values of data before SAC stimulation were as follows: *P* = 0.003 and *P*_c_ = 0.009 for healthy controls vs patients with active BD, and *P* = 0.007 and *P*_c_ = 0.021 for patients with inactive BD vs patients with active BD. After SAC stimulation, the statistical significance values for comparisons were as follows: *P* = 0.0032 and *P*_c_ = 0.010 for healthy controls vs patients with active BD and *P* = 0.0064 and *P*_c_ = 0.019 for patients with inactive BD vs patients with active BD. All data are representative of five experiments and are expressed as average ± standard deviation.

IL-27 mRNA expression of the two subunits was analyzed in PBMCs obtained from patients with active BD, patients with inactive BD and healthy controls. The mRNA expression of the unique subunit of IL-27 (IL-27p28) in patients with active BD (1.0 ± 0.7) was significantly lower than that in patients with inactive BD (2.4 ± 0.9) (*P* = 0.012) or healthy controls (2.2 ± 1.0; P = 0.032). No difference could be detected in the IL-27p28 mRNA level between patients with inactive BD and healthy controls. Also, no significant difference between the tested groups was detectable concerning the mRNA expression of the EBI3 subunit (Figure 1A).

### Decreased levels of IL-27 in serum and in supernatants of cultured PBMCs from patients with active BD

IL-27 levels were significantly lower in the serum from patients with active BD (955.5 ± 394.8 pg/ml) compared with patients with inactive BD (1,964.9 ± 1,885.8 pg/ml; *P* = 0.009) and healthy controls (2,608.9 ± 2,051.7 pg/ml; *P* < 0.001). No detectable difference was observed between patients with inactive BD and healthy controls (Figure 1B). We next analyzed IL-27 expression in PBMC culture supernatants and observed that unstimulated PBMCs from patients with active BD produced a lower level of IL-27 (585.7 ± 218.7 pg/ml) than unstimulated PBMCs from patients with inactive BD (1,041.2 ± 193.2 pg/ml; *P* = 0.021) and unstimulated PBMCs from healthy controls (1,103.5 ± 232.9 pg/ml; *P* = 0009). Stimulation with SAC increased the IL-27 production by PBMCs from BD patients and healthy controls. Moreover, the SAC-treated PBMCs in patients with active BD still produced a lower level of IL-27 (816.8 ± 163.5 pg/ml) compared to levels in patients with inactive BD (1,355.4 ± 259.4 pg/ml; *P* = 0.019) and levels in healthy controls (1,408.7 ± 314.9 pg/ml; *P* = 0.010) (Figure 1C). However, no difference was observed in the relative increase of IL-27, expressed as a percentage of controls, upon SAC stimulation in the three tested groups.

### Effect of recombinant IL-27 on IL-17 production and differentiation of Th17 cells in BD patients and healthy controls

**Figure 2:**
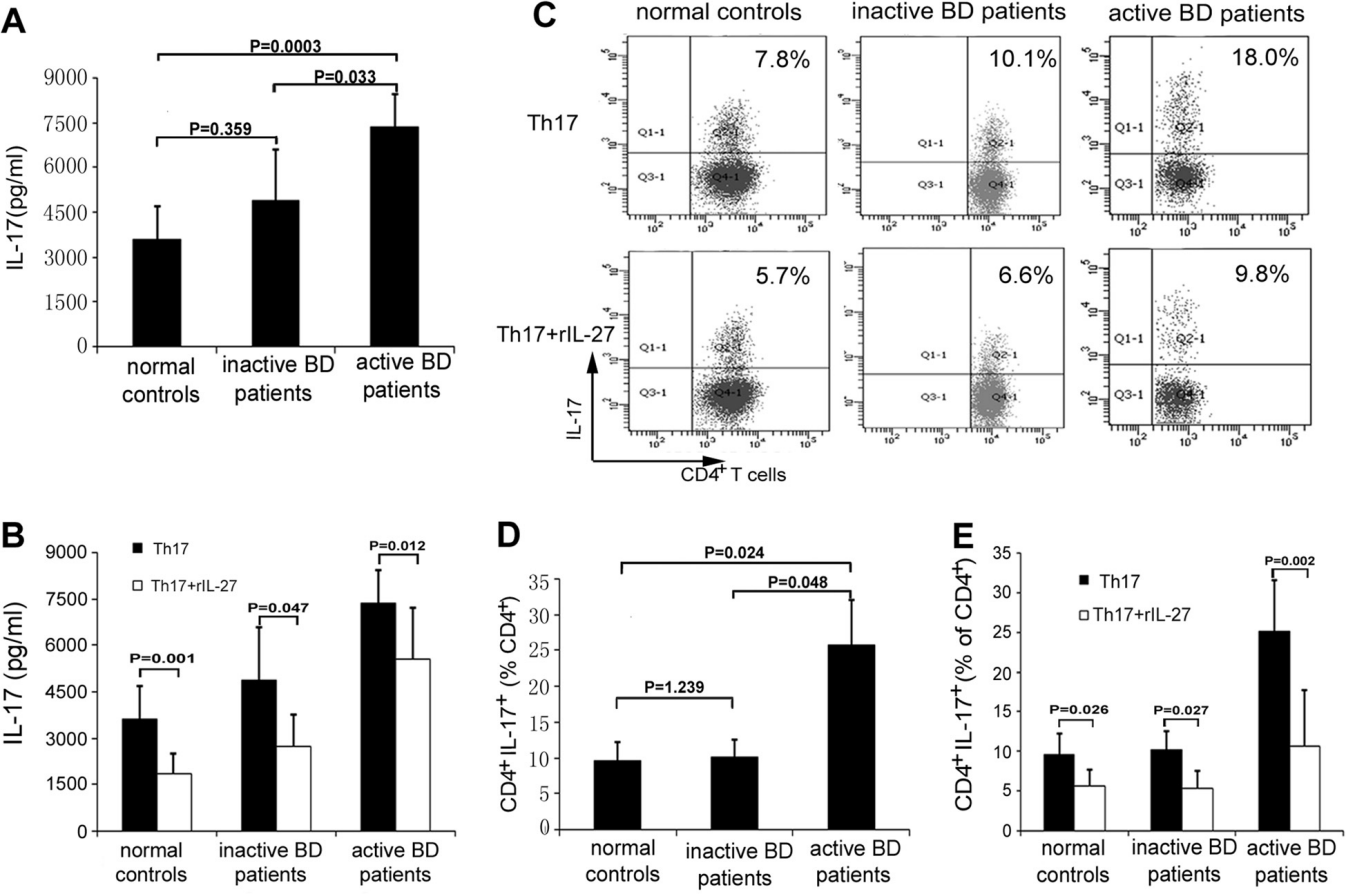
**Effect of recombinant interleukin 27 on interleukin 17 production and type 17 T helper cell differentiation in Behçet’s disease patients and healthy controls.** Naïve CD4^+^ T cells from patients with active Behçet’s disease (BD) (*n =* 8), patients with inactive BD (*n =* 8) and healthy controls (*n* = 8) were stimulated with or without recombinant interleukin 27 (rIL-27) (100 ng/ml) under type 17 T helper cell (Th17) polarizing conditions for 6 days. **(A)** and **(B)** IL-17 production in the culture supernatants of stimulated naïve CD4^+^ T cells was measured by enzyme-linked immunosorbent assay. **(C)** Intracellular staining of IL-17 was investigated by flow cytometric analysis. Representative flow cytometry plots for each group are shown. **(D)** and **(E)** Quantitative analysis of the percentage of IL-17-producing CD4^+^ T cells was carried out by paired-samples *t*-test for related samples and one-way analysis of variance for independent samples. All data are representative of five independent experiments and are expressed as average ± standard deviation.

Naïve CD4^+^ T cells can be differentiated *in vitro* into IL-17-producing effector (Th17) cells in a cytokine milieu containing anti-IFN-γ, anti-IL-4, anti-IL-1β, anti-IL-6 and anti-IL-23 antibodies [25,26]. In mouse studies, it has been shown that IL-27 plays a negative role in Th17 cell differentiation. To investigate the direct effect of rIL-27 on Th17 cell differentiation in humans, we cultured naïve CD4^+^ T cells from both BD patients and healthy controls under Th17 polarizing conditions with or without rIL-27. The results show that naïve CD4^+^ T cells from patients with active BD cultured under Th17 polarizing conditions produced more IL-17 (7,351.2 ± 1,110.6 pg/ml) than did naïve CD4^+^ T cells from patients with inactive BD (4,875.1 ± 1,729.6 pg/ml; *P* = 0.033) and healthy controls (3,597.0 ± 1,089.2 pg/ml; *P* = 0.0003). There was no detectable difference between patients with inactive BD and healthy controls concerning IL-17 production (Figure 2A). Exposure to rIL-27 led to significantly decreased IL-17 production by stimulated naïve CD4^+^T cells from patients with active BD (7,351.2 ± 1,110.6 vs 5,560.5 ± 1,647.5 pg/ml; *P* = 0.012), patients with inactive BD (4,875.1 ± 1,729.6 vs 2,722.5 ± 1,061.1 pg/ml; *P* = 0.047) and healthy controls (3,597.0 ± 1,089.2 vs 1,837.0 ± 684.5 pg/ml; *P* = 0.001). However, the inhibitory percentages did not differ among the tested groups (Figure 2B). To further investigate the role of rIL-27 in IL-17 production on a per-cell basis, intracellular staining of IL-17 was also performed in stimulated naïve CD4^+^ T cells. The results revealed that naïve CD4^+^ T cells from patients with active BD, when cultured under Th17 polarizing conditions, showed a significantly higher frequency of IL-17-producing CD4^+^ T cells in patients with active BD (25.8 ± 6.4) than did naïve CD4^+^ T cells from patients with inactive BD (10.2 ± 2.3; *P* = 0.048) or healthy controls (9.6 ± 2.6; *P* = 0.024). No significant difference was detectable between patients with inactive BD and healthy controls (Figures 2C and 2D). The addition of IL-27 showed significant inhibition of the frequency of IL-17-producing CD4^+^ T cells in patients with active BD (25.8 ± 6.4 vs 10.6 ± 7.2; *P* = 0.002), patients with inactive BD (10.2 ± 2.3 vs 5.3 ± 2.3; *P* = 0.027) and healthy controls (9.6 ± 2.6 vs 5.6 ± 2.0; *P* = 0.026) (Figures 2C and 2E). Moreover, rIL-27 inhibited the Th17 differentiation and IL-17 production in a dose-dependent manner (data not shown).

### Recombinant IL-27 inhibits human Th17 differentiation via the upregulation of IRF8

**Figure 3:**
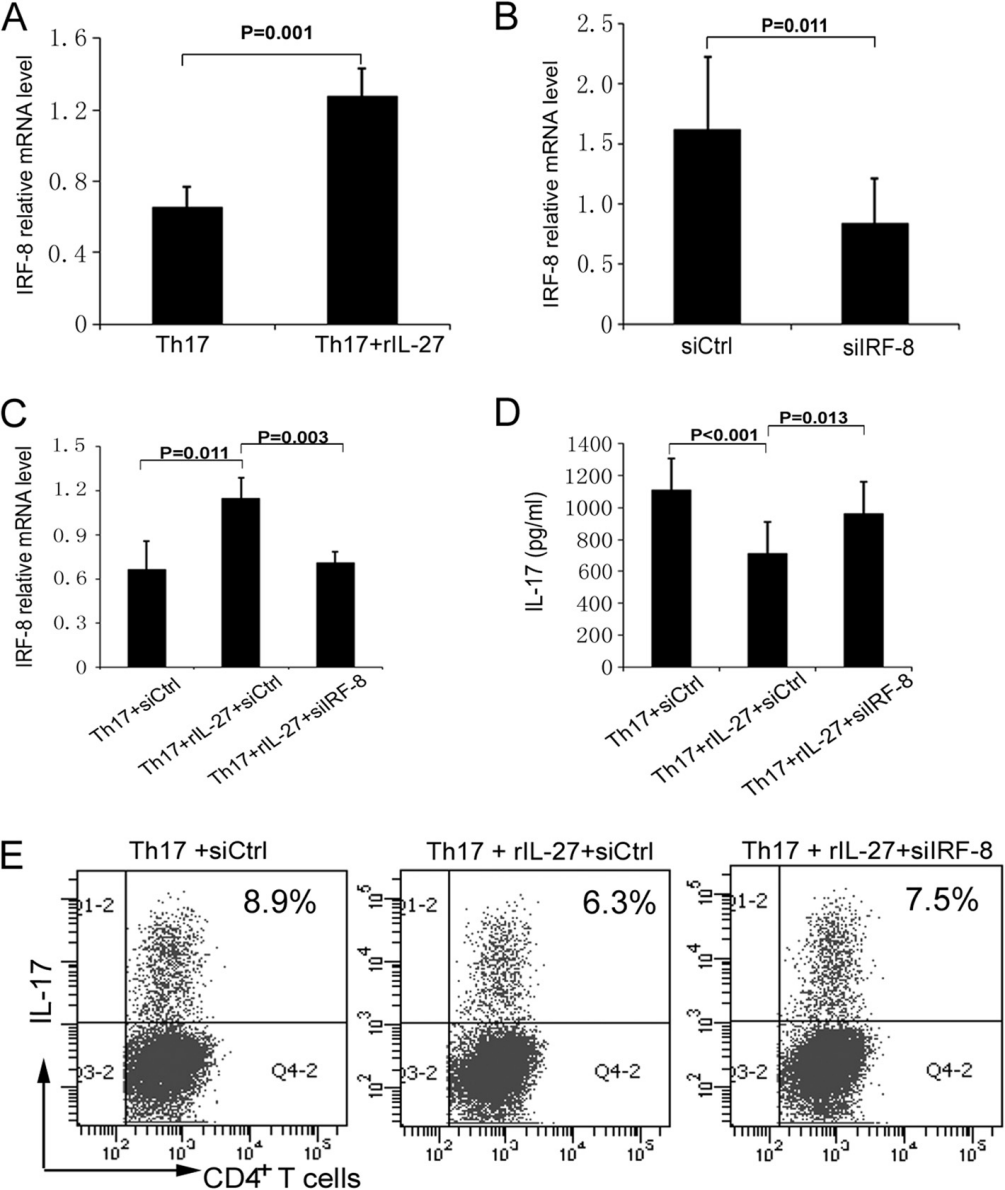
**Recombinant interleukin 27 inhibits human type 17 T helper cell differentiation via upregulation of IRF8. (A)** The expression of interferon regulatory factor 8 (*IRF8*) in naïve CD4^+^ T cells (*n* = 5) cultured under type 17 T helper cell (Th17) polarizing conditions in the presence or absence of interleukin 27 (IL-27) was detected by RT-PCR. **(B)** Real-time PCR analysis was carried out to determine *IRF8* expression in naïve CD4^+^ T cells from healthy controls (*n* = 5) nucleoporated with *IRF8*-specific small interfering RNA (siRNA; siIRF-8) or control siRNA (siCtrl) and then cultured under Th17 polarizing conditions. Naïve CD4^+^ T cells from healthy controls (*n* = 8) were nucleoporated with control siRNA or *IRF8*-specific siRNA and cultured with or without recombinant IL-27 (rIL-27) under Th17 polarizing conditions for another 48 hours. **(C)***IRF8* expression in these cells was detected by RT-PCR. **(D)** IL-17 production in the cell culture supernatants was measured by enzyme-linked immunosorbent assay. **(E)** Representative flow cytometry dot plots of intracellular IL-17 expression are shown. Paired-samples *t*-tests for related samples and analysis of variance for three different groups were used for statistical analysis. All of the data are representative of three independent experiments and are expressed as average ± standard deviation.

Recently, researchers have shown that IRF8 is involved in Th17 cell differentiation in mice [27]. The results described above show that rIL-27 inhibited Th17 differentiation in humans. Further experiments with RNA interference were performed to study whether IL-27 exerts its effect in humans by modulating IRF8. The results show that rIL-27 significantly upregulated IRF8 expression (0.7 ± 0.1 vs 1.3 ± 0.2; *P* = 0.001) (Figure 3A). Furthermore, we show that IRF8 siRNA successfully reduced the expression of IRF8 in naïve CD4^+^ T cells stimulated with IL-27 under Th17 polarizing conditions (1.3 ± 0.3 vs 0.6 ± 0.1; *P* = 0.002) (Figure 3B). The results of further experiments show that the suppressive activity of IL-27 on the production of IL-17 and the frequency of polarized Th17 cells was markedly decreased when we knocked down IRF8 expression (Figures 3C, 3D and 3E).

### Recombinant IL-27 inhibits expression of proinflammatory cytokines, but promotes release of anti-inflammatory cytokines by dendritic cells

**Figure 4:**
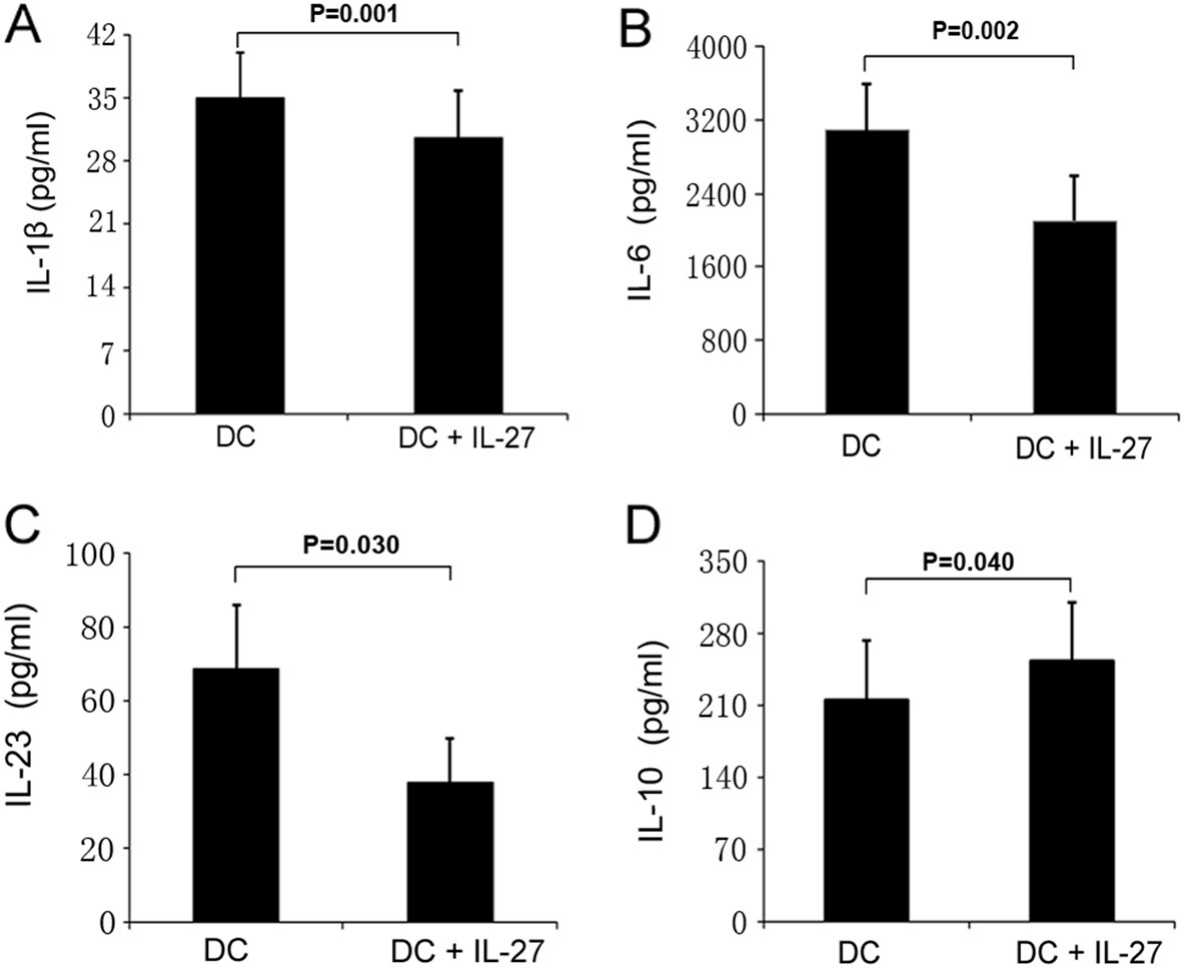
**Interleukin 27 inhibited production interleukin 1β, interleukin 6 and interleukin 23, but promoted production of interleukin 10, by dendritic cells. (A)** through **(D)** Interleukin 1β (IL-1β), IL-6, IL-23 and IL-10 in the supernatants of monocyte-derived dendritic cells (DCs) (monocyte-derived DCs from three healthy controls and two patients with active BD) stimulated with lipopolysaccharide in the absence or presence of IL-27 (100 ng/ml) for 48 hours were measured by enzyme-linked immunosorbent assay. Paired-samples *t*-tests for related samples were used for statistical analysis. All of the data are representative of three independent experiments with cells five donors and are expressed as average ± standard deviation.

DCs play a critical role in the initiation of immune responses against invading pathogens, as well as in the induction of central and peripheral tolerance [28]. It is well-established that the differentiation and development of T helper cell subsets are controlled by DCs. For example, DC-secreted IL-1β, IL-6 and IL-23 can induce differentiation of Th17 cells, whereas DC-secreted IL-10 initiates differentiation of Tr1 cells. To investigate the effect of rIL-27 on the production of cytokines by DCs, monocytes were cultured with GM-CSF and IL-4 for 6 days to generate immature DCs. These cells were then stimulated with LPS with or without rIL-27 for 24 hours. The results show that rIL-27 significantly inhibited the production of IL-1β (214.6 ± 58.5 vs 252.6 ± 56.5 pg/ml; *P* = 0.001), IL-6 (3,086.0 ± 726.6 vs 2,096.5 ± 746.2 pg/ml; *P* = 0.002) and IL-23 (68.5 ± 17.5 vs 38.0 ± 11.7; *P* = 0.030) by DCs, but induced a modest but significant increase in IL-10 production (34.8 ± 5.2 vs 30.5 ± 5.3; *P* = 0.040) (Figure 4).

### IL-27-treated dendritic cells inhibit Th1 and Th17 cell responses

**Figure 5:**
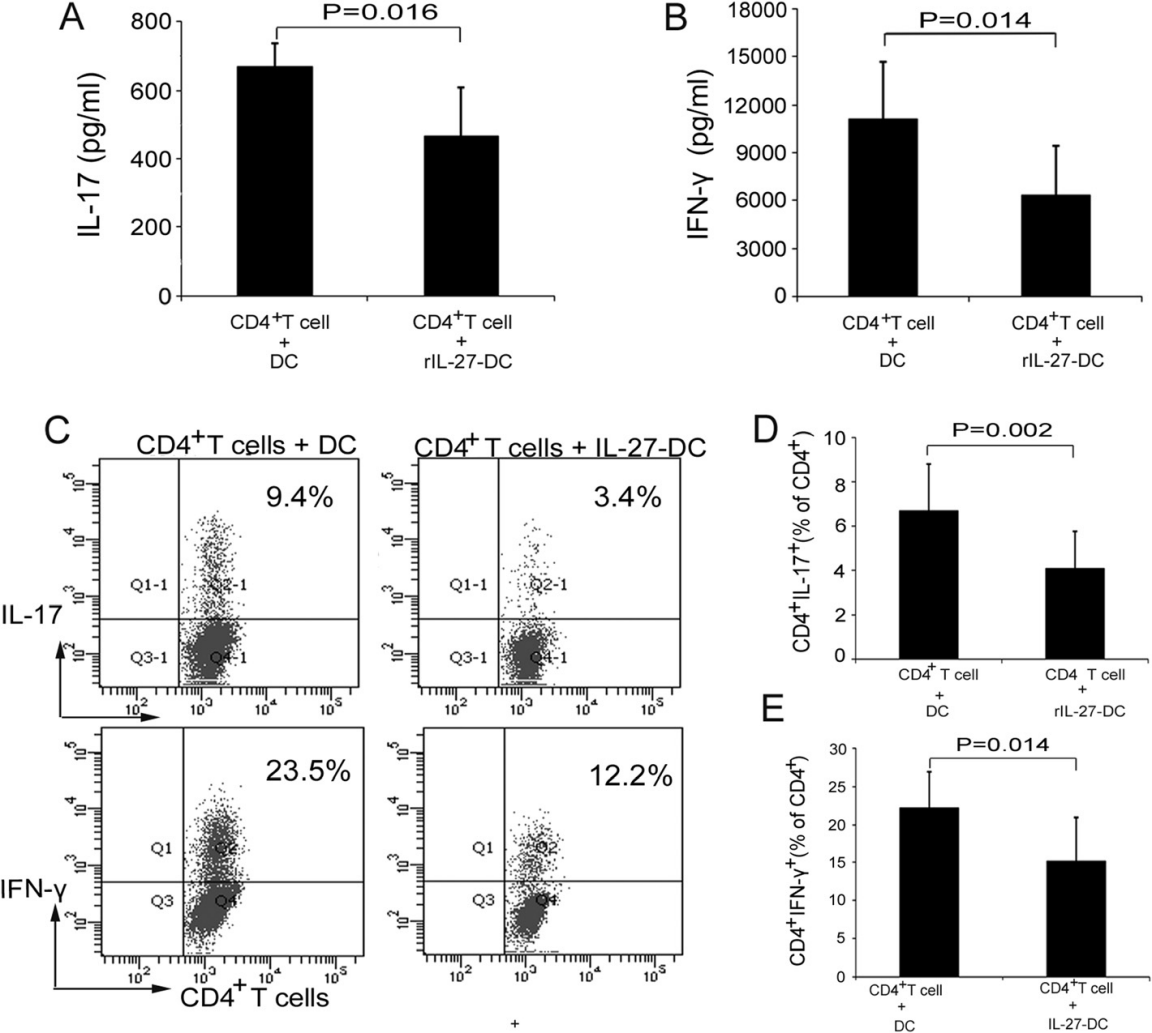
**Interleukin 27–treated dendritic cells inhibited types 1 and 17 T helper cell response. (A)** and **(B)** Allogeneic CD4+ T cells were cocultured with dendritic cells (DCs) or interleukin 27 (IL-27)–treated DCs (monocyte-derived DCs from four healthy controls and four patients with active BD) at a ratio of 5:1 for 4 days. Interferon γ (IFN-γ) and IL-17 production in the cell culture supernatants was determined by enzyme-linked immunosorbent assay. **(C)** Intracellular expression of IFN-γ and IL-17 was analyzed by flow cytometry. Representative flow cytometry dot plots for each group are shown. **(D)** and **(E)** Quantitative analysis of the frequency of IL-17-producting and IFN-γ-producing CD4+ T cells was carried out by paired-samples *t*-tests for related samples. All of the data are representative of three independent experiments and are expressed as average ± standard deviation.

We further assessed the role of rIL-27-treated DCs in the T-cell response. The aforementioned rIL-27-treated DCs were washed and then cultured with CD4^+^ T cells for 3 days. The results show that the release of IL-17 and IFN-γ into the culture supernatant was inhibited when the T cells were cultured with IL-27-treated DCs (670.0 ± 65.0 vs 467.8 ± 139.2 pg/ml (*P* = 0.016) and 11,132.8 ± 3,575.6 vs 6,323.7 ± 3,120.5 pg/ml (*P* = 0.014), respectively) (Figures 5A and 5B). Our flow cytometric analysis shows that coculturing CD4^+^ T cells with IL-27-treated DCs resulted in decreased frequency of IL-17- and IFN-γ-producing T cells compared to LPS-treated control DCs (6.7 ± 2.1 vs 4.1 ± 1.7 (*P* = 0.002) and 22.2 ± 4.8 vs 15.2 ± 5.8 (*P* = 0.014), respectively) (Figures 5C, 5D and 5E).

## Discussion

In the present study, we provide evidence that decreased expression of IL-27 may be involved in the pathogenesis of Behçet’s disease. The inhibition of IL-27 on Th17 differentiation might be mediated via the IRF8 pathway. Our *in vitro* data support the hypothesis that IL-27 affects both Th1 and Th17 cell differentiation by an effect on DCs.

Our results confirm those obtained in earlier studies in experimental autoimmune disease models in which IL-27 was shown to play a role as a master negative regulator in both EAE and EAU by suppressing the development of Th1 and Th17 cells [7,14,29,30] and promoting the development of Tr1 cells [31]. IL-27 can be expressed only as a heterodimeric cytokine composed of the subunits p28 (IL-27p28) and the EBI3 in humans; however, it can also be expressed as a monomer containing only the p28 subunit in mice [32]. IL-27 signals via a receptor complex composed of the IL-6 receptor gp130 and the IL-27 receptor α chain. The subunit IL-27p28 is unique to IL-27, and its regulated transcription is necessary for the secretion of this cytokine, whereas EIB3 is a constructive subunit that can associate with IL-12p35 to form IL-35 [33]. In our present study, we found a decreased level of IL-27p28 mRNA and IL-27 protein in patients with active BD compared to that in patients with inactive BD and healthy controls. The reasons why BD patients produce insufficient amounts of IL-27 remain an intriguing question that deserves further study.

Our results are consistent with the hypothesis that decreased IL-27 expression correlates with uveitis activity in BD patients. Whether our findings are specific to ocular BD patients and whether they can be extrapolated to patients without eye involvement and patients with other autoinflammatory diseases remain to be clarified. We recently reported that decreased IL-27 serum levels were also found in an autoimmune uveitis entity, Vogt-Koyanagi-Harada syndrome, but not in a group of patients with active acute anterior uveitis [34]. These findings suggest that the role of IL-27 might be restricted to certain intraocular inflammation entities. Further studies are needed to investigate the role of IL-27 in other immune-mediated uveitis entities that we have not yet investigated, including sarcoid uveitis and birdshot choroidopathy. Our results are in line with those described in previous reports in which there was a lower serum expression of IL-27 in patients with active systemic lupus erythematosus, multiple myeloma and multiple sclerosis [35-38].

Shen *et al*. recently reported increased levels of IL-27 in patients with Behçet’s disease [39]. The reasons for this discrepancy are not clear, but they could relate to large differences in the BD patient population in the two studies. The patients included in the Shen *et al*. study were recruited from a rheumatology department, and therefore arthritis and related systemic manifestations may have been the main clinical features of these patients, although they were also reported to have active uveitis. Another possibility is that there may have been differences in the treatment regimens used in their patient populations. We found decreased IL-27p28 mRNA expression in the PBMCs and decreased IL-27 protein expression in the serum and supernatants of cultured PBMCs in patients with active BD. Whether decreased IL-27 expression also occurs at the site of inflammation is not yet clear and requires analysis of cells from the vitreous or the cerebrospinal fluid.

The association between decreased IL-27 expression and active intraocular inflammation in BD indicates that IL-27 is involved in BD development. As Th17 cells have a critical role in the pathogenesis of BD, we first studied the role of rIL-27 in the differentiation of Th17 cells. The results show that rIL-27 significantly inhibited the frequency of IL-17-producing CD4^+^ T cells and IL-17 production these cells were cultured under Th17 polarizing conditions. These findings validate the negative regulatory effect of rIL-27 on Th17 cell differentiation, and the data are consistent with those reported earlier in studies conducted by other researchers [40] in which IL-27 was also shown to inhibit IL-17 secretion under Th17 polarizing conditions. Recently, Geri *et al*. reported increased expression of IL-21 in the serum of patients with active BD and that the increased IL-21 promoted Th17 differentiation [41]. Their results suggest that cytokines modulating Th17 cell differentiation might exert a critical role in the pathogenesis of BD. Moreover, we found an increased frequency of IL-17-producing CD4^+^ T cells and IL-17 production by naïve T cells cultured under Th17 polarizing conditions in patients with active BD. These results suggest that a large amount of IL-17-producing CD4^+^ T cells and increased IL-17 expression as observed in active BD might play a role in the appearance of active intraocular inflammation in these patients. Further studies are needed to investigate why naïve CD4^+^ T cells from patients with patients with active BD developed into more Th17 cells and whether these polarized Th17 cells, in addition to expressing more IL-17, produced a higher level of other cytokines, such as IFN-γ, GM-CSF, IL-2 and IL-10. A study comparing other cytokine expression patterns with the IL-27 response might be worthwhile to show the interaction between various inflammatory pathways.

A number of mechanisms involved in the suppressive effect of IL-27 on Th17 cell differentiation have been proposed. Some researchers have reported that IL-27 can inhibit Th17 differentiation through a signal transducer and activator of transcription 1 (STAT1)–dependent mechanism [7]. Other researchers have also suggested that the suppressive effect might be due to the induction of Tr1 cells by IL-27 [13]. Researchers in a recent study reported that IRF8 is a novel intrinsic transcriptional inhibitor of Th17 cell differentiation [27]. In our present study, by using the RNA interference method, we found that the inhibitory effect of rIL-27 on human Th17 cell differentiation was mediated by upregulation of the expression of IRF8. Studies of STAT1 phosphorylation are necessary to further analyze the intracellular pathways and the exact role of IRF8 in this process.

DCs play a critical role in the initiation of the immune response against invading pathogens and in the induction of immune tolerance [28,42]. Autoinflammatory diseases, including BD, are thought to arise from an aberrant response against environmental triggers whereby DCs play an important role in determining which subpopulation of helper T cells is activated [28]. In view of the important role of DCs in T-cell activation, we focused on the influence of IL-27 on the function of DCs. The results show that rIL-27 significantly inhibited the expression of proinflammatory cytokines, such as IL-1β, IL-6 and IL-23. The results of further experiments show that CD4^+^ T cells cocultured with IL-27-treated DCs had a decreased capacity to secrete IFN-γ and IL-17 compared to coculture with untreated DCs. These findings are in agreement with those reported by others [40,43]. Moreover, we observed that rIL-27-treated DCs produced slightly more IL-10 and that they inhibited the frequency of IFN-γ-producing and IL-17-producing CD4^+^ T cells. All of these results, taken together, support an important role of IL-27 as a negative modulator of the Th1 and Th17 cell response. Earlier studies by our group revealed a higher percentage of IFN-γ-producing and IL-17-producing CD4^+^ T cells in active ocular BD patients [8]. This upregulated Th1 and Th17 response may be attributed to the decreased expression of IL-27 observed in our present study. Future studies of the effect of IL-27 administration on the degree of ocular inflammation in BD patients may provide further fundamental evidence to support this hypothesis.

## Conclusion

The results of our study show that there is lower expression of IL-27 in patients with active BD. Recombinant IL-27 was able to inhibit Th17 differentiation in both BD patients and healthy controls. Our results also provide evidence that the negative regulatory effect of IL-27 on Th1 and Th17 cells was mediated via DCs and suggest the involvement of the IRF8 pathway in the suppressive effect of IL-27 on Th17 differentiation. All these studies support the hypothesis that a decreased expression of IL-27 is associated with active intraocular inflammation in BD, and an upregulation of this regulatory cytokine may provide a novel strategy for the treatment of this disease.

## Abbreviations

APC: Antigen-presenting cell; BD: Behçet’s disease; DC: Dendritic cell; EAE: Experimental autoimmune encephalomyelitis; EAU: Experimental autoimmune uveitis; *EBI3*: Epstein–Barr virus-induced gene 3; IL: Interleukin; IRF8: Interferon regulatory factor 8; STAT1: Signal transducer and activator of transcription 1; Tr1: Type 1 regulatory T cell.

## Competing interests

The authors declare that they have no competing interests.

## Authors’ contributions

CKW was responsible for the study conception and design, data collection and analysis, manuscript writing and critical revision of the manuscript. YT, ZY and YZ were responsible for data collection and participated in drafting the manuscript. AK participated in study design and critical revision of the manuscript. PZY was responsible for the study conception and design, manuscript writing and critical revision of the manuscript. All authors read and approved the final manuscript.

## Supplementary Material

Additional file 1 **Detailed demographics of patients with active or inactive Behçet’s disease. Table S1.** Detailed demographics of the patients with active Behçet’s disease. **Table S2.** Detailed demographics of the patients with inactive Behçet’s disease. Click here for file
