# Supplementary material for: Decreased interleukin 27 expression is associated with active uveitis in Behçet’s disease
Source: Arthritis Res Ther. 2014 May 28;16(3):R117. doi: 10.1186/ar4570 (PMC4095700; doi:10.1186/ar4570)
Supplement: Additional file 1 — Detailed demographics of patients with active or inactive Behçet’s disease. Table S1. Detailed demographics of the patients with active Behçet’s disease. Table S2. Detailed demographics of the patients with inactive Behçet’s disease. [file ar4570-S1.doc]

Additional file 1: Table S1 The detailed demographics of the active BD patients

| **Patients number** | **Age ranges（years）** | | **System corticosteroids** | **Ocular Manifestation** | | | | | | | | | | | **Visual Acuity** | | | | **Main Extraocular Findings** | | | | | | | |  | |  |
| --- | --- | --- | --- | --- | --- | --- | --- | --- | --- | --- | --- | --- | --- | --- | --- | --- | --- | --- | --- | --- | --- | --- | --- | --- | --- | --- | --- | --- | --- |
|  |  |  |  | **KPs** | | **Aqueous flare** | | | **Aqueous cells** | | **Vitreous cells** | | **Retinitis or Retinal vasculitis** | | **od** | | **os** | | **Oral ulcer** | | **Genital ulcer** | | **Skin lesions** | | **Positive pathergy test** | | **arthritis** | |  |
| **1** | 20-24 | No drugs | | | **+** | | **++** | | | **+** | | **+** | | **+** | | **HM** | | **HM** | | **+** | | **-** | | **+** | | **-** | | **+** | |
| **2** | 40-44 | No drugs | | | **+** | | **+** | | | **+** | | **+** | | **+** | | 0.01 | | 0.03 | | **+** | | **-** | | **+** | | **+** | | **+** | |
| **3** | 20-24 | No drugs | | | **++** | | **+** | | | **+++** | | **+** | | **+** | | 0.06 | | 0.3 | | **+** | | **-** | | **+** | | **-** | | **+** | |
| **4** | 30-34 | No drugs | | | **++** | | **+** | | | **+++** | | **-** | | **+** | | 0.05 | | 0.1 | | **+** | | **+** | | **+** | | **-** | | **-** | |
| **5** | 24-30 | No drugs | | | **++** | | **++** | | | **+++** | | **+** | | **+** | | 0.08 | | 0.15 | | **+** | | **+** | | **+** | | **-** | | **-** | |
| **6** | 34-40 | No drugs | | | **+** | | **+** | | | **+** | | **+** | | **+** | | 0.2 | | 0.15 | | **+** | | **-** | | **+** | | **+** | | **-** | |
| **7** | 34-40 | No drugs | | | **++** | | **+++** | | | **++** | | **+** | | **+** | | 0.06 | | 0.05 | | **+** | | **+** | | **+** | | **+** | | **-** | |
| **8** | 34-40 | No drugs | | | **++** | | **+** | | | **+** | | **-** | | **+** | | **HM** | | 0.12 | | **+** | | **-** | | **+** | | **-** | | **-** | |
| **9** | 34-40 | No drugs | | | **++** | | **+** | | | **+++** | | **+** | | **+** | | 0.08 | | 0.06 | | **+** | | **+** | | **-** | | **-** | | **-** | |
| **10** | 30-34 | No drugs | | | **+** | | **+** | | | **+** | | **+** | | **+** | | 0.7 | | 1.2 | | **+** | | **-** | | **+** | | **-** | | **-** | |
| **11** | 30-34 | No drugs | | | **+++** | | | **+++** | | **++** | | **-** | | **+** | | 0.02 | | 0.03 | | **+** | | **+** | | **+** | | **-** | | **-** | |
| **12** | 40-44 | No drugs for 20 days | | | **++** | | **+++** | | | **+** | | **+** | | **+** | | 0.04 | | HM | | **+** | | **+** | | **+** | | **-** | | **-** | |
| **13** | 20-24 | No drugs | | | **++** | | **++** | | | **+** | | **+** | | **+** | | 0.5 | | 0.4 | | **+** | | **-** | | **-** | | **-** | | **+** | |
| **14** | 40-44 | No drugs | | | **+** | | **++** | | | **+++** | | **+** | | **+** | | 0.1 | | 0.2 | | **+** | | **-** | | **+** | | **+** | | **+** | |
| **15** | 50-54 | No drugs for 15 days | | | **++** | | **++** | | | **+++** | | **+** | | **+** | | **HM** | | LP | | **+** | | **+** | | **+** | | **-** | | **-** | |
| **16** | 30-34 | No drugs | | | **++** | | **+** | | | **++** | | **-** | | **+** | | FC | | 0.15 | | **+** | | **-** | | **+** | | **-** | | **-** | |
| **17** | 34-40 | No drugs for 20 days | | | **+** | | **+** | | | **+** | | **+** | | **+** | | 0.1 | | 0.08 | | **+** | | **+** | | **+** | | **-** | | **+** | |
| **18** | 40-44 | No drugs for 15 days | | | **++** | | **+++** | | | **++** | | **-** | | **+** | | 0.1 | | 0.1 | | **+** | | **-** | | **+** | | **-** | | **-** | |
| **19** | 24-30 | No drugs for 1 month | | | **++** | | **+** | | | **+** | | **+** | | **+** | | 0.4 | | LP | | **+** | | **+** | | **+** | | - | | **-** | |

HM：Hand Movement， LP：Light Perception； FC：figure counting

Additional file 1: Table S2 The detailed demographics of the inactive BD patients

| **Patients number** | **Age ranges**  **（years）** | **Ocular Manifestation** | | | | | **Visual Acuity** | | | **Main Extraocular Findings*** | | | | | |  |
| --- | --- | --- | --- | --- | --- | --- | --- | --- | --- | --- | --- | --- | --- | --- | --- | --- |
|  |  | **KPs** | **Aqueous flare** | **Aqueous cells** | **Vitreous cells** | **Retinitis or Retinal vasculitis** | | **od** | **os** | | **Oral ulcer** | **Genital ulcer** | **Skin lesions** | **Positive pathergy test** | **arthritis** | |
| **1** | 40-44 | **-** | **-** | **-** | **-** | **-** | | LP | NLP | | **+** | **+** | **+** | **-** | **-** | |
| **2** | 24-30 | **-** | **-** | **-** | **-** | **-** | | 0.02 | 1 | | **+** | **+** | **+** | **-** | **+** | |
| **3** | 30-34 | **-** | **-** | **-** | **-** | **-** | | 1 | HM | | **+** | **+** | **-** | **+** | **-** | |
| **4** | 34-40 | **-** | **-** | **-** | **-** | **-** | | 1 | 0.04 | | **+** | **+** | **+** | **-** | **+** | |
| **5** | 24-30 | **-** | **-** | **-** | **-** | **-** | | 0.6 | 0.06 | | **+** | **-** | **+** | **-** | **-** | |
| **6** | 34-40 | **-** | **-** | **-** | **-** | **-** | | 0.1 | 0.15 | | **+** | **-** | **+** | **-** | **-** | |
| **7** | 40-44 | **-** | **-** | **-** | **-** | **-** | | 0.25 | LP | | **+** | **+** | **+** | **-** | **+** | |
| **8** | 30-34 | **-** | **-** | **-** | **-** | **-** | | 0.6 | 0.3 | | **+** | **+** | **+** | **-** | **-** | |
| **9** | 40-44 | **-** | **-** | **-** | **-** | **-** | | 0.04 | 0.3 | | **+** | **+** | **+** | **+** | **-** | |
| **10** | 34-40 | **-** | + | **-** | **-** | **-** | | 0.03 | 0.02 | | **+** | **+** | **+** | **+** | **-** | |
| **11** | 24-30 | **-** | **-** | **-** | **-** | **-** | | 0.04 | 1.5 | | **+** | **-** | **+** | **+** | **-** | |
| **12** | 24-30 | **-** | **-** | **-** | **-** | **-** | | 0.01 | 0.08 | | **+** | **+** | **-** | **-** | **-** | |
| **13** | 24-30 | **-** | **-** | **-** | **-** | **-** | | 0.05 | 0.2 | | **+** | **+** | **+** | **-** | **-** | |
| **14** | 44-50 | **-** | **-** | **-** | **-** | **-** | | NLP | FC | | **+** | **+** | **+** | **+** | **-** | |
| **15** | 30-34 | **-** | **-** | **-** | **-** | **-** | | 0.5 | 0.4 | | **+** | **-** | **+** | **-** | **+** | |
| **16** | 34-40 | **-** | **-** | **-** | **-** | **-** | | 0.04 | FC | | **+** | **-** | **+** | **-** | **-** | |
| **17** | 24-30 | **-** | + | **-** | **-** | **-** | | 0.3 | 0.1 | | **+** | **+** | **+** | **-** | **-** | |
| **18** | 24-30 | **-** | ± | **-** | **-** | **-** | | 0.8 | 1.2 | | **+** | **+** | **-** | **-** | **-** | |
| **19** | 34-40 | **-** | **-** | **-** | **-** | **-** | | 0.4 | 0.05 | | **+** | **-** | **+** | **-** | **-** | |
| **20** | 44-50 | **-** | **-** | **-** | **-** | **-** | | 0.2 | 0.16 | | **+** | **+** | **+** | - | **-** | |

* previously present but absent at blood sampling
